# Exposure to IQOS ads and reduced exposure claims, and association with perceived risk from COVID-19 on IQOS purchase and use intentions: results from a web-based survey

**DOI:** 10.3389/fpubh.2023.1307484

**Published:** 2024-01-10

**Authors:** Akshika Sharma, Brian Fix, Andrew Hyland, Maansi Bansal-Travers, Amanda Quisenberry, Richard O’Connor

**Affiliations:** ^1^Department of Psychiatry, Yale School of Medicine, New Haven, CT, United States; ^2^Department of Health Behavior, Roswell Park Comprehensive Cancer Center, Buffalo, NY, United States

**Keywords:** heated tobacco products, IQOS, marketing, reduced exposure claims, COVID-19

## Abstract

**Introduction:**

IQOS was authorized to be marketed as a reduced exposure product by the Food and Drug Administration in October 2020 during the global COVID-19 pandemic. Those who smoke may be more sensitive to reduced exposure marketing claims and may have an increased inclination toward IQOS use. We evaluated the likelihood of trying and purchasing IQOS as a function of exposure to ads, product appeal, and COVID-19 risk perceptions using the original IQOS ads with reduced exposure marketing.

**Method:**

An online cross-sectional survey recruited 604 US adults (18–45 years), both who smoke and do not smoke. Participants saw one of the six randomly assigned IQOS ads with or without reduced exposure claims, and they answered questions about product appeal and likelihood to try and purchase IQOS. Generalized linear models were used to examine associations.

**Results:**

A per unit increase in product appeal was associated with a greater likelihood of purchasing (B = 0.17, 95% CI: 0.15–0.18) and trying IQOS (B = 0.16, 95% CI = 0.14–0.18). Current smokers and former e-cigarette users reported greater intentions to try IQOS than never-smokers and never e-cigarette users, respectively. Likelihood to purchase IQOS was associated with greater confidence in not contracting COVID-19 (B = 0.11, 95% CI: 0.01–0.21). No significant differences were observed between different ad conditions. Current (B = −0.34, 95% CI = −0.50-(−0.19)) and former (B = −0.92, 95% CI = −0.15-(−0.68)) cigarette smokers who were someday e-cigarette users reported less intentions to purchase IQOS than never e-cigarette users. However, never smokers who were someday (B = 0.58, 95% CI = 0.27–0.89; B = 0.68, 95% CI = 0.39–0.98) and former e-cigarette (B = 0.38, 95% CI = 0.15–0.61) users reported greater intentions to purchase and try IQOS, respectively.

**Discussion:**

IQOS may have a higher product appeal, especially for those who currently smoke and those who have lower risk perceptions from COVID-19. Among never smokers, those who currently use or have used e-cigarettes in the past may be more receptive to IQOS marketing. The data are informative for potential trends in the use of IQOS in the future and may have implications for marketing regulations of heated tobacco products (HTPs).

## 1 Introduction

Tobacco companies continue to engage users’ interests in and demand for less harmful alternatives to commercially available cigarettes through innovations, such as heated tobacco products (HTPs) (1). HTPs first arrived in US markets in the 1980s with the introduction of Premier (1988), followed by Eclipse (1996), Accord (1998), and Revo (2014) (2). Although these early designs were withdrawn from markets, modern HTPs (since 2014) have been marketed and widely adopted in countries like Japan (3). In the United States, Phillip Morris International (PMI) received authorization from the Food and Drug Administration (FDA) to market their HTP product IQOS in 2019 (4, 5). The word ‘IQOS’ is not an acronym (6) (Supplementary Figure 1). IQOS uses a technology that heats a cigarette-like stick in a device below the point of combustion (350°F), releasing aerosols that can then be inhaled by the user (7). Furthermore, in October 2020, IQOS was authorized by FDA to include reduced exposure claims in their marketing (8), specifically the statements ‘*The IQOS system heats tobacco but does not burn it*’—‘*This significantly reduces the production of harmful and potentially harmful chemicals*’ referred henceforth in this manuscript as claim 1 and ‘*Scientific studies have shown that switching completely from conventional cigarettes to the IQOS system significantly reduces your body’s exposure to harmful or potentially harmful chemicals*’, referred to as claim 2. However, there remain uncertainties around the perception of these claims by tobacco users and non-users once the product is in the marketplace. A specific concern is that the design and marketing claims of IQOS may influence non-users into believing that it has reduced health risks compared to smoking (4, 9, 10).

Furthermore, in 2020, the world experienced the COVID-19 pandemic, where data suggested that cigarette smokers had an increased susceptibility to poorer outcomes (hospitalization and mortality) if they contracted coronavirus (11–13). Additionally, pandemic-induced lockdowns, financial losses, and stress influenced smoking behaviors both positively and negatively. There were reports of increased smoking in response to stress and isolation, but also cessation attempts in response to fear of sickness (14–17). Continued smoking (18) and e-cigarette use (19, 20) were associated with a greater perception of getting sick with COVID-19 and resulted in smoking cessation in individuals. In such a scenario, reduced exposure alternatives to cigarettes could become increasingly salient to existing users. Thus, the intersection of consumer response to reduced exposure marketing claims in the context of an ongoing pandemic and its attendant risk perceptions is a critical area to explore. The objective of this study is to assess the association between exposure to IQOS advertisements with and without reduced exposure claims and consumers’ intentions to use and purchase IQOS. We also evaluated an association of COVID-19 risk perceptions with purchase and use intention. Our primary hypothesis is that the participants assigned to IQOS ad conditions containing reduced exposure claims will report higher product appeal for IQOS and intentions to use IQOS (intentions to purchase and try) than those assigned to control ad conditions without reduced exposure claims. Our secondary hypothesis is that the participants reporting a greater product appeal for IQOS after viewing the ad will report greater intentions to use IQOS and that COVID-19-related worry will influence the intentions of using IQOS in the near future.

## 2 Methods

A 20-min web-based survey recruited 18–45 years-old US residents from two online platforms (Amazon Mechanical Turk and Prime Panels) in December 2021 and January 2022. Amazon Mechanical Turk, popularly known as Mturk, is a large crowdsourcing platform that has been widely used for research. Participants on Mturk self-select themselves for participation in a study based on its eligibility criteria and monetary rewards, whereas Prime Panels are participant pools drawn from commercial panels based on specific eligibility criteria (21). After providing informed consent, participants were asked about their demographics, current tobacco product use, and their awareness of, or experience with HTPs. Participants also responded to their risk perceptions and worry about getting COVID-19 infection.

### 2.1 Ad conditions

**Figure 1:**
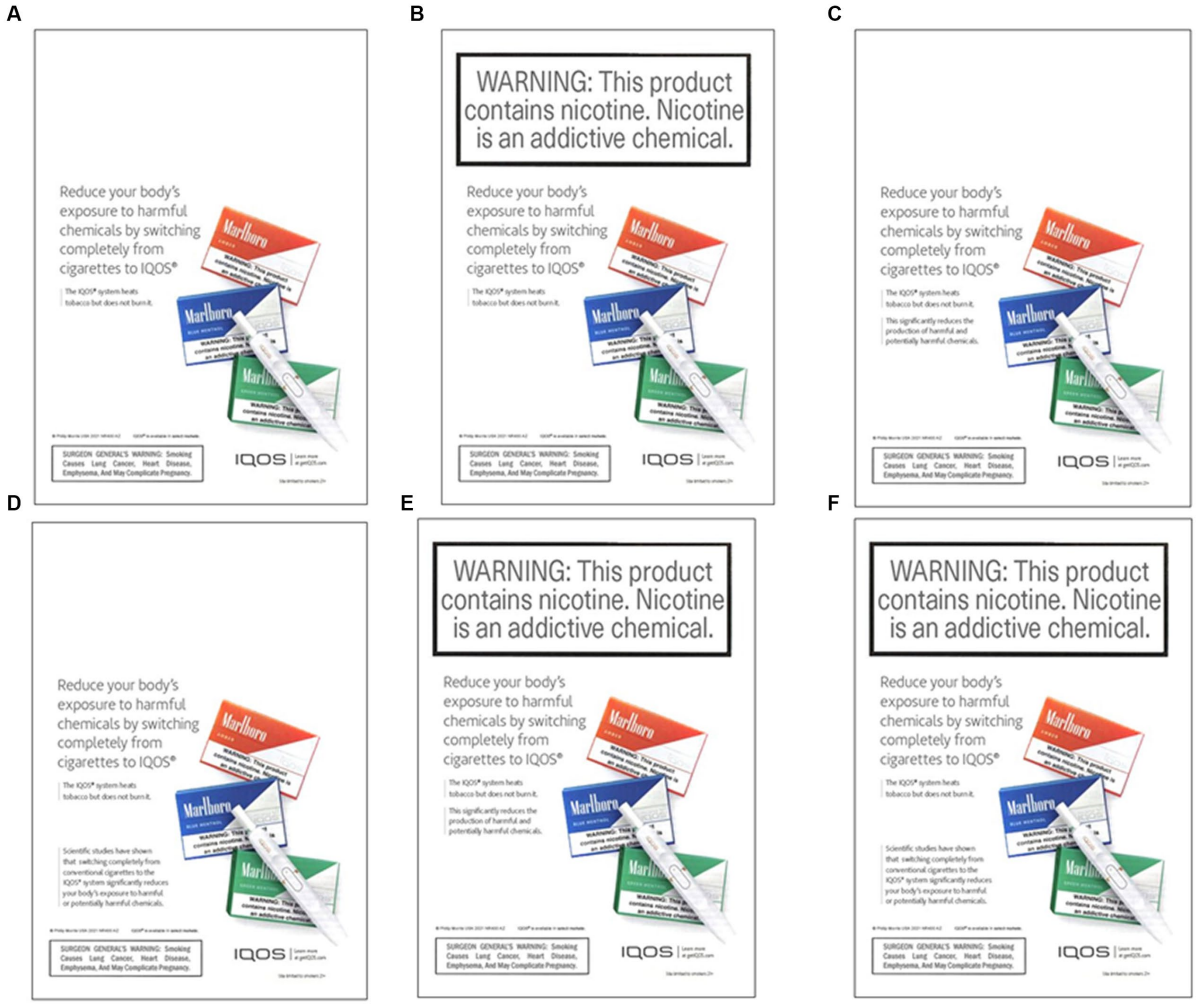
Ad conditions were randomly assigned to participants. **(A)** Ad only, which contained a picture of the product along with the introductory statement and surgeon general’s warning, **(B)** ad + health warning (HW), which contained additional HW stating ‘*this product contains nicotine. Nicotine is an addictive chemical’,* **(C)** ad + claim 1, **(D)** ad+ claim 2, **(E)** ad+ HW + claim 1, **(F)** ad + HW + claim 2.

Finally, participants were randomly assigned to view one of the six IQOS advertisements (ad condition A–condition F). The IQOS ad sample was obtained from trinkets and trash marketing materials (22). Based on the research question for the current study, the ad was modified to create six different ad conditions (Figure 1). A—ad only, which contained a picture of the product along with the introductory statement and surgeon general’s warning; B—ad + health warning (HW), ad only contained additional HW stating ‘*this product contains nicotine. Nicotine is an addictive chemical’;* C*—*ad + claim 1; D—ad+ claim 2; E—ad+ HW + claim 1; F—ad + HW + claim 2. A and B were used as control conditions to compare against test conditions C–F (Figure 1).

Participants were subsequently asked questions about the most appealing part of the ad they saw, IQOS product appeal, and intentions to try and purchase IQOS. The study procedures were approved by the Institutional Review Board at Roswell Park Comprehensive Cancer Center (protocol I-1389821).

## 3 Measures

### 3.1 Demographics and tobacco use

All participants answered questions about their age, gender, race, ethnicity, education, and annual income. Tobacco use was measured by asking a series of different questions, for example, ‘*Have you smoked at least 100 cigarettes in your entire life?’ (yes/no)* and ‘*Do you now smoke cigarettes?’ (everyday, someday, and not at all).* Ever use and current use of electronic nicotine delivery systems (ENDS) was also assessed with the same response options as cigarette smoking. Finally, current smoking and e-cigarette use statuses were derived as four categorical variables (everyday, someday, former, and never), respectively.

### 3.2 Introduction of HTPs

All participants, regardless of their smoking status, were shown a picture of an HTP (Supplementary Figure 1) with a brief description and asked about their familiarity or use of the product. Immediately following the description, participants were asked, ‘*thinking about heated tobacco products, which of the following statements BEST applies to you?’* The response options included *(a) I have never heard of heated tobacco products before today, (b) I have heard of heated tobacco products but have never tried them, (c) I have tried heated tobacco products but do not use them anymore, and (d) I currently use heated tobacco products.* For analysis, the variable was grouped into the following two categories: those who reported being unaware (those who chose response options *a* and not *b, c*, or *d*) and those who were aware of HTPs (those who chose response options *b, c*, or *d*).

### 3.3 Response to ad condition and intentions to use

The participants were randomly assigned to view one of the six IQOS ads (Figure 1). Their perceptions of the ad and product were assessed. They were asked, ‘*Of the advertisement of IQOS that you just saw, which part you find the most appealing?’.* The response options included *‘(1) picture of the device, (2) name of the device, (3) description of the product, (4) surgeon general’s warning, (5) health warning, and (6) claim that it produces less toxicants than cigarette*’. Furthermore, they were asked, ‘*Overall, on a scale of 0–10, how appealing is this product?’*. Response options ranged from *not at all appealing (0) to extremely appealing (10)*. The main outcomes were assessed by asking questions such as the likelihood to try, ‘*If you were offered an opportunity to try IQOS for free, how likely would you be to do so?’* Purchase intentions were measured by asking*, ‘How likely are you to purchase IQOS in the next 12 months?’.* Response options ranged on an 11-point scale from *no chance (0) to practically certain (10).*

### 3.4 COVID-19 risk perceptions

Participants’ perception of COVID-19-related risk was measured by asking them to respond to the statement ‘*I am confident that I will not get COVID-19 novel coronavirus’*. The response options ranged on a 5-point scale from ‘*Strongly disagree to Strongly agree, & I have already tested positive for COVID-19′*. Those who reported already testing positive for COVID-19 (*n* = 25) were removed from the analysis when specifically looking at related associations. Participants reported their worry about COVID-19 infection by responding to ‘*How worried are you about getting COVID-19 novel coronavirus?*’ responses were measured on a 5-point scale from ‘*Not at all worried to extremely worried*’.

## 4 Data analysis

Frequency distribution was used to assess distribution by demographics (age, sex, race, and ethnicity) and reported tobacco use status. One-way ANOVA was used to identify differences in ad outcomes (product appeal, intentions to purchase, and try IQOS) by ad conditions, as well as between smoking and e-cigarette use status and ad outcomes. Independent *t*-tests were used to report a bivariate association between COVID-19 risk perceptions and outcomes. For outcome, measures such as product appeal, intentions to purchase, and try IQOS were used as continuous variables. Chi-square tests were used to observe associations between smoking and e-cigarette use status and appeal for ad components. Data distribution for main outcomes was observed for skewness and kurtosis, and generalized linear regression models (GLM) were used to assess associations while adjusting for skewness of data. Final GLM regression models were built with product appeal as a main predictor and intentions to purchase IQOS (Table 1) and intentions to try IQOS (Table 2) as dependent variables, respectively. Co-variates used in models included the demographics (age, sex, race, ethnicity, education, and income), recruitment platforms, current smoking status, current e-cigarette use status, worry about contacting COVID-19, and confidence about not contracting COVID-19. A sub-group analysis was carried out by smoking status at the time of study by restricting the sample to current, former, and never-smokers, respectively, and using the same predictors and co-variates as main models. Variable ‘worry for contracting COVID-19’ was dichotomized as ‘*no or less worry’* (those who chose 0 = not at all, 1 = a little, 2 = somewhat) and ‘*greater worry’* (4 = very, 5 = extremely), and ‘confidence for not contracting COVID-19’ was dichotomized as ‘*disagree* (those who chose 1 = strongly disagree, 2 = disagree) and ‘*agree’* (3 = agree, 4 = strongly agree). All analyses were carried out using IBM SPSS 28.0.

**Table 1:** Generalized linear model for intentions to purchase IQOS in the next 12 months.

Variable name		B-coefficient	Std. error	95% CI		value of *p*
				Lower	Upper	
Product appeal		0.17	0.01	0.15	0.18	**<0.001**
Recruitment platform	Prime panel	0.17	0.05	0.06	0.27	**0.002**
	Mturk	Ref	–	–	–	–
Randomized ad condition	F	−0.03	0.08	−0.18	0.12	0.70
	E	−0.04	0.08	−0.19	0.11	0.56
	D	−0.05	0.08	−0.20	0.10	0.49
	C	0.05	0.07	−0.09	0.19	0.48
	B	−0.06	0.07	−0.21	0.08	0.39
	A	Ref	–	–	–	–
Current smoking status	Everyday smoker	0.46	0.06	0.33	0.58	**<0.001**
	Someday smoker	0.47	0.07	0.32	0.61	**<0.001**
	Former smoker	−0.04	0.08	−0.18	0.11	0.61
	Never smoker	Ref	–	–	–	–
Current ENDS use	Everyday user	0.05	0.08	−0.11	0.21	0.56
	Someday user	−0.04	0.06	−0.17	0.09	0.55
	Former user	0.09	0.06	−0.03	0.20	0.14
	Never user	Ref	–	–	–	–
Worry about contracting COVID-19	More worry	0.08	0.05	−0.01	0.18	0.09
	No or less worry	Ref	–	–	–	–
Confident of not contracting COVID-19	Agreeing	0.11	0.05	0.01	0.21	**0.03**
	Disagreeing	Ref	–	–	–	–
Age	18–20	Ref	–	–	–	–
	21–30	0.19	0.17	−0.32	0.36	0.92
	31–40	−0.72	0.17	−0.41	0.27	0.68
	41–45	−0.006	0.18	−0.36	0.35	0.98
Sex	Male	Ref	–	–	–	–
	Female	0.90	0.05	−0.00	0.18	0.06
Race and ethnicity	Non-Hispanic White individuals	Ref	–	–	–	–
	Non-Hispanic Black individuals	−0.11	0.07	−0.24	0.03	0.12
	Hispanic individuals	−0.01	0.07	−0.14	0.12	0.90
	Others	−0.14	0.08	−0.29	0.02	0.08
Income	<$20,000	Ref	–	–	–	–
	$20,000 to $34,999	0.14	0.08	−0.02	0.29	0.09
	$35,000 to $49,999	0.14	0.07	0.003	0.27	**0.04**
	$50,000 to $74,999	0.05	0.08	−0.11	0.21	0.54
	$75,000 to $99,999	0.00	0.08	−0.16	0.16	1.00
	≥ $100,000	−0.82	0.08	−0.24	0.07	0.30
Education	High school or less	Ref	–	–	–	–
	Post-High school	−0.12	0.12	−0.36	0.11	0.31
	Some college	−0.21	0.07	−0.35	−0.08	**0.003**
	College graduate and post-graduate	−0.15	0.06	−0.27	−0.02	**0.02**

OR, Odds ratio; CI, Confidence interval.

**Table 2:** Generalized linear model for intentions to try IQOS if offered for free.

Variable name		B-coefficient	Std. error	95% CI		value of *p*
				Lower	Upper	
Product appeal		0.16	0.01	0.14	0.18	**<0.001**
Recruitment platform	Prime panel	0.13	0.06	−0.12	0.10	0.81
	Mturk	Ref	–	–	–	–
Randomized ad condition	F	−0.16	0.08	−0.31	0.03	0.06
	E	−0.06	0.07	−0.22	0.09	0.44
	D	−0.07	0.08	−0.23	0.08	0.36
	C	0.02	0.08	−0.13	0.16	0.83
	B	−0.17	0.08	−0.32	−0.02	**0.03**
	A	Ref	–	–	–	–
Current smoking status	Everyday smoker	0.45	0.06	0.33	0.58	**<0.001**
	Someday smoker	0.41	0.07	0.26	0.55	**<0.001**
	Former smoker	0.08	0.08	−0.07	0.24	0.28
	Never smoker	Ref	–	–	–	–
Current ENDS use	Everyday user	−0.02	0.09	−0.19	0.15	0.80
	Someday user	0.21	0.07	0.07	0.34	**0.003**
	Former user	0.08	0.06	−0.04	0.19	0.21
	Never user	Ref	–	–	–	–
Worry about contracting COVID-19	More worry	0.04	0.05	−0.06	0.14	0.45
	No or less worry	Ref	–	–	–	–
Confident of not contracting COVID-19	Agreeing	0.06	0.05	−0.04	0.16	0.22
	Disagreeing	Ref	–	–	–	–
Age (years)	18–20	Ref	–	–	–	–
	21–30	0.06	0.18	−0.29	0.42	0.72
	31–40	0.02	0.18	−0.33	0.38	0.89
	41–45	−0.02	0.19	−0.39	0.34	0.90
Sex	Male	Ref	–	–	–	–
	Female	0.06	0.05	−0.04	0.16	0.22
Race and ethnicity (*N* = 604)	Non-Hispanic White individuals	Ref	–	–	–	–
	Non-Hispanic Black individuals	−0.17	0.07	−0.31	−0.03	**0.02**
	Hispanic individuals	−0.01	0.07	−0.15	0.13	0.90
	Others	−0.16	0.08	−0.32	−0.01	**0.04**
Annual income (US dollars) (*N* = 604)	<$20,000	Ref	–	–	–	–
	$20,000 to $34,999	0.14	0.08	−0.03	0.30	−0.10
	$35,000 to $49,999	0.20	0.07	0.06	0.34	**0.00**
	$50,000 to $74,999	0.18	0.08	0.01	0.34	**0.04**
	$75,000 to $99,999	0.18	0.09	0.02	0.35	**0.03**
	≥ $100,000	0.11	0.08	−0.05	0.27	0.19
Education (*N* = 604)	High school or less	Ref	–	–	–	–
	Post-High school	−0.04	0.12	−0.29	0.20	0.73
	Some college	−0.03	0.07	−0.17	0.11	0.69
	College graduate and post-graduate	−0.10	0.07	−0.23	0.03	0.15

OR, Odds ratio; CI, Confidence interval.Bold numbers indicate significant differences with α < 0.05.

## 5 Results

### 5.1 Demographics and tobacco use

Overall, we recruited a total of 604 participants from both Prime Panels and Mturk. More than 49% of the participants were between 31 and 40 years old, with approximately 51% men and 49% women. Non-Hispanic white individuals constituted most of the sample with 67% representation, and individuals identifying as non-Hispanic Black and Hispanic each represented approximately 12% of the sample. In total, 54% of the participants reported being college graduates and post-graduates, and 55% reported an annual income of more than $50,000.

Of the sample, 67% reported they had smoked at least 100 cigarettes in their lifetime; 31% reported currently smoking cigarettes everyday, 21% somedays, and 14% not at all. 68% of participants reported having ever used ENDS, even one or two times; of these, 13% reported current everyday use, 31% reported someday use, and 23% reported being former users. Although 44% of users reported using only e-cigarettes, 20% said they had used more than one electronic nicotine product (Table 3).

**Table 3:** Demographic and tobacco use characteristics of survey participants.

S.no.	Variable name		Overall *N* (%)
1.	Age (years) (*N* = 604)	18–20	12 (2.0)
		21–30	204 (33.8)
		31–40	298 (49.3)
		41–45	90 (14.9)
2.	Sex (*N* = 604)	Male	306 (50.7)
		Female	295 (48.8)
		Non-Binary and Transgender	3 (0.5)
4.	Race and ethnicity (*N* = 604)	Non-Hispanic White individuals	402 (66.6)
		Non-Hispanic Black individuals	73 (12.1)
		Hispanic individuals	72 (11.9)
		Others	56 (9.3)
5.	Annual income (US dollars) (*N* = 604)	<$20,000	112 (18.5)
		$20,000 to $34,999	72 (11.9)
		$35,000 to $49,999	78 (12.9)
		$50,000 to $74,999	130 (21.5)
		$75,000 to $99,999	74 (12.3)
		≥ $100,000	130 (21.5)
		No response	8 (1.3)
6.	Education (*N* = 604)	High school or less	126 (20.9)
		Post-High school	24 (4.0)
		Some college	126 (20.9)
		College graduate and post-graduate	328 (54.3)
7.	100-lifetime cigarettes (*N* = 604)	Yes	397 (65.7)
		No	207 (34.3)
8.	Current smoking status (*N* = 397)	Everyday	189 (31.3)
		Somedays	124 (20.5)
		Not at all	84 (13.9)
9.	e-cigarette ever use (*N* = 604)	Yes	408 (67.5)
		No	194 (32.1)
		Do not know	2 (0.3)
10.	Do you now use electronic products? (*N* = 408)	Everyday	81 (13.4)
		Somedays	189 (31.3)
		Not at all	138 (22.8)
11.	Type of e-product ever used (*N* = 406)	e-cigarette only	263 (43.5)
		Other e-products	20 (3.3)
		More than one e-product	123 (20.4)

Bold numbers indicate significant differences with α < 0.05.

### 5.2 Awareness or use of HTPs

When asked about their experience with HTPs, approximately 56% reported that they had never heard of HTPs, while 28% chose ‘*I have heard of heated tobacco products but have never tried them*’, 7% chose ‘*I have tried heated tobacco products but do not use them anymore*’, and approximately 10% chose ‘*I currently use heated tobacco products*’. Based on cell sizes, the responses were dichotomized into those unaware of HTPs (55.8%) and those who reported being aware of them (44.2%). Those who reported being aware of HTPs reported a higher product appeal (*t* (df) = 8.0 (602), *p* = 0.14) and a greater likelihood to try IQOS (*t* (df) = 7.32 (602), *p* < 0.001) than those who were unaware of HTPs.

### 5.3 COVID-19 risk perceptions

Participants responded to the statement *‘I am confident I will not contract COVID-19*’. In a bivariate analysis, those who agreed with the above statement reported significantly higher intentions to try IQOS (*t* (df) = 4.73 (577), *p* < 0.001) than those who disagreed with the statement. Participants further responded to the question, ‘*How worried are you about contracting COVID-19?*’. Those who reported ‘greater worry’ reported a significantly greater likelihood to purchase IQOS (*t* (df) = 4.74 (602), *p* < 0.001) than those reporting ‘no or less worry’.

### 5.4 Factors associated with response to ad condition and likelihood of use

**Figure 2:**
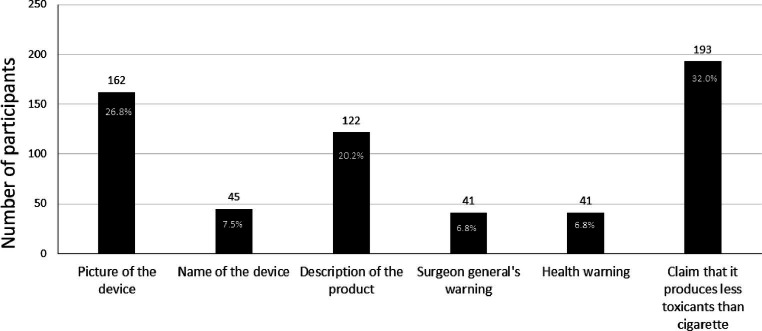
The most appealing part of the IQOS ad as reported by participants.

Those exposed to different ad conditions did not differ significantly in responses to product appeal and intentions to try or purchase IQOS (Supplementary Table 1). When asked about the most appealing part of the ad, 32% of participants reported the reduced exposure claims, whereas 27% found the picture of the IQOS device most appealing. The description of the product was appealing to 20% of the participants, and 7% reported each name of the device, HW, and surgeon general’s warning as most appealing (Figure 2). Significantly greater appeal for reduced exposure claims, picture of the device, and name of the device was reported by current (everyday and someday) smokers as compared with former or never-smokers (chi-square = 36.43, value of *p* <0.01). Additionally, a greater proportion of former users of e-cigarettes responded to reduced exposure claims and the picture of the device as more appealing than current e-cigarette users. However, the difference was non-significant. Approximately 28% of participants in ad condition A (ad only) and 35% in ad condition B (ad+ HW only) reported reduced exposure claims to be the most appealing to them.

Those endorsing currently smoking reported significantly higher means for product appeal (F (df) = 44.68 (3), *p* < 0.001), intentions to purchase (F (df) = 98.26 (3), *p* < 0.001), and intentions to try (F (df) = 89.06 (3), *p* < 0.001) IQOS than those reporting never and former smoking. However, former e-cigarette users reported a greater product appeal (F (df) = 11.16 (3), *p* < 0.001), intentions to purchase (F (df) = 19.13 (3), *p* < 0.001), and intentions to try (F (df) = 13.08 (3), *p* < 0.001) IQOS as compared with everyday, someday, and never users.

Generalized linear regression models were used to assess independent associations with appeal, intent to try, and intent to purchase. The first model assesses associations between product appeal and the likelihood of purchasing IQOS in the next 12 months. The model was adjusted for demographics, recruitment platform, smoking status, and e-cigarette use status, as well as COVID-19 risk perception variables (Table 1). A per unit increase in product appeal was associated with greater odds of purchasing IQOS (B = 0.17, 95% CI: 0.15–0.18). Everyday smokers (B = 0.46, 95% CI = 0.33–0.58) and someday smokers were more likely (B = 0.47, 95% CI = 0.32–0.61) to purchase IQOS than never-smokers. Those recruited via Prime Panels showed a greater likelihood than those recruited via Mturk (B = 0.17, 95% CI = 0.06–0.27) for purchasing IQOS. A greater confidence in not contracting COVID-19 was associated with a greater purchase likelihood (B = 0.11, 95% CI = 0.01–0.21). An annual income of $35,000–$49,999 (B = 0.14, 95% CI = 0.003–0.27) was positively, and college (B = −0.21, 95% CI = −0.35-(−0.08)) and higher education (B = −0.15, 95% CI = −0.27−(−0.02)) were negatively associated with purchase likelihood.

The second model represents the association between product appeal and intentions to try IQOS. Per unit increase in product appeal resulted in a greater likelihood to try IQOS (B = 0.16, 95% CI = 0.14–0.18). Those who were assigned to ad condition B (ad + HW only) reported lower odds of trying IQOS than Condition A (ad only) (B = −0.17, 95% CI = −0.32-(−0.02)). Everyday smokers (B = 0.45, 95% CI = 0.33–0.58) and someday smokers (B = 0.41, 95% CI = 0.26–0.55) reported a greater likelihood to try IQOS than never-smokers. However, someday e-cigarette users reported a greater likelihood to try IQOS (B = 0.21, 95% CI = 0.07–0.34) than never users of e-cigarettes. Those who identified as Non-Hispanic Black (B = −0.17, 95% CI = −0.31-(−0.03)) and as other racial group (B = −0.16, 95% CI = −0.32-(−0.01)) reported lower intentions to try IQOS as compared with NH White individuals. Those reporting annual income from $35,000 up to $99,999 ((B = 0.20, 95% CI = 0.06–0.34); (B = 0.18, 95% CI = 0.01–0.34); (B = 0.18, 95% CI = 0.02–0.35)) also reported a higher likelihood to try IQOS than those with income under $20,000. No significant differences in intentions to try were observed based on the recruitment platform and worry about contracting COVID-19 (Table 2).

A sub-group analysis by smoking status showed, among current smokers, a higher product appeal (B = 0.13, 95% CI = 0.11–0.15), being a female (B = 0.13, 95% CI = 0.03–0.23), and annual income greater than $20,000 ($20,000–$34,999, B = 0.27, 95% CI = 0.10–0.44; $35,000–$49,999, B = 0.28, 95% CI = 0.11–0.45; $50,000–$74,999, B = 0.30, 95% CI = 0.13–0.46; $75,000–$99,999, B = 0.34, 95% CI = 0.15–0.54; ≥$100,000, B = 0.33, 95% CI = 0.16–0.51) were positively associated with intentions to purchase IQOS. However, someday e-cigarette use (B = −0.34, 95% CI = −0.50−(−0.19)), college, and higher education (B = −0.15, 95% CI = −0.29–0.01) were negatively associated with intentions to purchase. Intentions to try IQOS in current smokers were positively associated with higher product appeal (B = 0.09, 95% CI = 0.07–0.11), being a female (B = 0.10, 95% CI = 0.01–0.18), and annual income of $35,000–$49,999 (B = 0.16, 95% CI = 0.01–0.31); (Supplementary Tables 2, 3). Among former smokers, greater intentions to purchase were positively associated with higher product appeal (B = 0.11, 95% CI = 0.07–0.14), the confidence of not contracting COVID-19 (B = 0.27, 95% CI = 0.02–0.52), and annual income of above $100,000 (B = 0.32, 95% CI = 0.01–0.63) than income under $20,000. However, it was negatively associated with someday (B = −0.92, 95% CI = -0.15−(−0.68)) e-cigarette use and some college (B = −0.35, 95% CI = 0.60−(−0.09)) and higher education (B = −0.22, 95% CI = −0.43−(−0.01)) than high school or less. Intentions to try IQOS among former smokers were positively associated with higher product appeal (B = 0.16, 95% CI = 0.11–0.21), being a female (B = 0.36, 95% CI = 0.09–0.64), and greater confidence of not contracting COVID-19 (B = 0.60, 95% CI = 0.24–0.95), and negatively with an assignment to ad condition D (ad+ claim 2) (B = −0.48, 95% CI = −0.84−(−0.12)), recruitment via Prime Panels (B = −0.41, 95% CI = −0.74−(−0.09)) than Mturk, and ages 41 years and above (B = −0.40, 95% CI = −0.80−(−0.12)) as compared to age 21–30 years (Supplementary Tables 4, 5). Finally, among never-smokers, a greater intention to purchase IQOS was positively associated with greater product appeal (B = 0.16, 95% CI = 0.13–0.19), someday e-cigarette use (B = 0.58, 95% CI = 0.28–0.89), recruitment via Prime Panels (B = 0.29, 95% CI = 0.06–0.52), and confidence of not contracting COVID-19 (B = 0.22, 95% CI = 0.01–0.43). Intentions to try IQOS among never smokers were associated with higher product appeal (B = 0.18, 95% CI = 0.15–0.21), being someday (B = 0.68, 95% CI = 0.39–0.98), former e-cigarette users (B = 0.38, 95% CI = 0.15–0.61), and recruitment via Prime Panels (B = 0.29, 95% CI = 0.07–0.51); (Supplementary Tables 6, 7).

## 6 Discussion

Our study aims to evaluate a critical association between ads and claims for novel tobacco-based product IQOS and various factors that may influence uptake or switch between products in a sample of adults and young adults. Our results indicate a greater appeal among participants for components of IQOS ads such as claims that it produces less toxicants, a picture of the device, and a description of the product. Perhaps unsurprisingly, greater appeal was observed in current smokers than in non-smokers, indicating they might be more susceptible to marketing IQOS with such claims. We also observed a greater appeal for claims and pictures of devices in former e-cigarette users (although non-significant), which may be indicative of their interest in trying innovative design-based products close in resemblance to e-cigarettes. A significantly greater likelihood to try IQOS was also reported by someday e-cigarette users, which may indicate a greater inclination toward IQOS in those who are experimenting with e-cigarettes or may be looking for a less harmful product. Authorization of reduced exposure claims granted to IQOS may have an influence on increasing its appeal for current or former tobacco users. This finding resonates well with the concern that has been raised by many researchers regarding the misinterpretation of reduced exposure as reduced risk (4, 23). However, of those who reported reduced exposure claims to be most appealing, 33% had seen an ad without a claim. This could be an artifact of the questionnaire containing this response option but could also refer to respondents interpreting other elements of the ad (such as pictures, descriptive text, or the HW) as ‘claims.’ For example, all ads contained a statement, ‘*The IQOS system heats tobacco but does not burn it’,* which could have been interpreted by respondents as a claim. The responses to the ads might have also been influenced by confusion between different products, such as mistaking IQOS with cigarettes or e-cigarettes, owing to a close similarity between heat sticks and cigarettes and the design of devices (24).

A significantly greater likelihood to try IQOS and purchase IQOS was observed in those who reported a greater product appeal. This association is rather expected (25). Our results are somewhat similar to a study that showed an association between attention to promotional content in IQOS ads (a proxy for appeal) and susceptibility to use (26). Receptivity to advertisements of tobacco products and consequential increased interest in the use of products have also been previously reported and emphasized (27, 28). In our study, ad believability, product appeal, likelihood to try IQOS, and likelihood of purchasing IQOS did not seem to differ among participants in different ad conditions. However, we observed a lower likelihood of trying among those who were assigned to conditions with ad and HW (C2), which may signify a greater receptivity for HWs in advertisements. The GLM analysis shows a significantly greater likelihood among everyday and someday smokers for trying and purchasing IQOS when compared with never-smokers. These results are in line with those reported by similar studies (29, 30). This finding reinforces the requirement for clear communication regarding differences between reduced exposure and reduced risk when advertising for IQOS, as well as the significance of completely switching from cigarettes to IQOS for harm reduction. This may help to avoid dual use or uptake by new users, as observed before with e-cigarettes. Furthermore, the long-term health risks from the use of HTPs are still unknown, and they might be comparable to damage caused by other tobacco products (31). We observed lower intentions to try among Non-Hispanic Black and other racial groups than Non-Hispanic White individuals. This highlights the need for more research to assess impact of IQOS uptake on existing health disparities among racial and ethnic groups. Reporting of middle to high income was positively associated with a greater likelihood of trying IQOS. It may be explained by the innovative design of the product that may appeal more to those seeking expensive-looking devices. However, higher education was inversely associated with intentions to purchase.

Among smoking sub-groups, we observed comparative responses for current and former smokers. We found that someday e-cigarette use in these groups was negatively associated with intentions to try and purchase IQOS. It may be due to various reasons, such as current smokers who are someday e-cigarette users, e-cigarettes may be serving a temporary switch from cigarettes, and they may not be inclined to add a new product to the mix. However, former smokers who are someday e-cigarette users may be using e-cigarettes to deal with nicotine cravings and prevent relapse to smoking or may be looking to transition out of nicotine use entirely. We also observed women among current and former smokers reporting greater intentions to try and purchase IQOS, which is in contrast with previous reports showing men have greater reduced harm perceptions and an increased intention to use HTPs than women (32, 33). Considering the highly fashionable design of IQOS, it can be presumed to gather attention from especially female sex in the future. However, among never smokers who were someday or former e-cigarette users, we observed greater intentions to purchase and try IQOS. This may indicate a potentially higher likelihood of new users uptaking IQOS and has critical importance for its regulation.

In a bivariate assessment between main outcomes and COVID-19 risk perceptions, we observed a significantly higher likelihood to try and purchase IQOS in those reporting a greater confidence of not contracting COVID-19 but also those reporting a greater worry about contracting COVID-19. These results resonate with the change in smoking and vaping behaviors reported as an influence of the COVID-19 pandemic (14, 34). This may be because of participants’ thrill-seeking attitudes, as well as the self-perceived risk of COVID-19; their tobacco product use might be a representation of their risk-taking behavior. However, in a multinomial model, we only observed a significant association between confidence in not contracting COVID-19 and a greater likelihood of purchasing.

Our findings are suggestive of greater uptake of IQOS by current smokers, as well as a sub-sample of never-smokers who may have tried e-cigarettes before and who may find the product more appealing. Our study adds to the limited existing knowledge about the potential impact of FDA authorization for IQOS reduced exposure claims and the user groups that might be more interested in IQOS. The results are informative for public health officials to better understand the expected trends in the use of HTPs in coming the years (35). While IQOS sales were discontinued in the US in November 2021 due to a patent conflict, Philip Morris International recently purchased the US sales rights back from Altria with intentions to relaunch the product (36). If and when IQOS returns to US markets, the study results suggest a need for close post-marketing surveillance to assess consumer understanding of claims to minimize dual use by current smokers or uptake by new users.

## Data availability statement

The raw data supporting the conclusions of this article will be made available by the authors, without undue reservation.

## Ethics statement

The studies involving humans were approved by the Institutional Review Board of Roswell Park Comprehensive Cancer Center. The studies were conducted in accordance with the local legislation and institutional requirements. The participants provided their written informed consent to participate in this study.

## Author contributions

AS: Conceptualization, Data curation, Formal analysis, Investigation, Methodology, Project administration, Software, Writing – original draft, Writing – review & editing. BF: Data curation, Investigation, Methodology, Visualization, Writing – review & editing. AH: Methodology, Resources, Supervision, Writing – review & editing. MB-T: Supervision, Visualization, Writing – review & editing. AQ: Investigation, Supervision, Writing – review & editing. RO’C: Conceptualization, Data curation, Funding acquisition, Investigation, Methodology, Project administration, Resources, Supervision, Writing – review & editing.
