# Supplementary material for: Exposure to IQOS ads and reduced exposure claims, and association with perceived risk from COVID-19 on IQOS purchase and use intentions: results from a web-based survey
Source: Front Public Health. 2024 Jan 10;11:1307484. doi: 10.3389/fpubh.2023.1307484 (PMC10805831; doi:10.3389/fpubh.2023.1307484)
Supplement: Supplementary file 1 [file Table_1.DOCX]

| Random ad condition | Ad outcome measures | | |
| --- | --- | --- | --- |
|  | Product appeal for IQOS  M (SD)* | Intentions to purchase IQOS  M (SD)* | Intentions to try IQOS  M (SD)* |
| Ad only | 6.48 (±3.3) | 4.89 (±3.7) | 6.78 (±4.0) |
| Ad + health warning (HW) | 6.72 (±3.2) | 5.03 (±3.8) | 6.16 (±4.0) |
| Ad + claim 1 | 6.89 (±3.2) | 5.62 (±3.7) | 7.05 (±3.5) |
| Ad + claim 2 | 6.46 (±3.2) | 5.09 (±3.5) | 6.72 (±3.9) |
| Ad + HW + claim 1 | 6.70 (±3.02) | 5.09 (±3.6) | 6.89 (±3.7) |
| Ad + HW + claim 2 | 6.85 (±3.3) | 5.70 (±3.6) | 6.78 (±3.8) |

Supplementary table 1. Bivariate analysis characterizing responses to ad outcome measure across ad conditions. M= Mean, SD= Standard Deviation. *No significant mean differences for product appeal, intentions to purchase and intentions to use were observed between ad conditions.

| Variable name | | B-coefficient | Std. error | 95% CI | | p-value |
| --- | --- | --- | --- | --- | --- | --- |
|  |  |  |  | Lower | Upper |  |
| Product appeal | | 0.13 | 0.01 | 0.11 | 0.15 | **<0.001** |
| Recruitment platform | Prime panel | 0.05 | 0.05 | -0.05 | 0.16 | 0.31 |
|  | Mturk | Ref | - | - | **-** | - |
| Randomized ad condition | F | 0.11 | 0.08 | -0.04 | 0.26 | 0.15 |
|  | E | 0.04 | 0.08 | -0.12 | 0.19 | 0.65 |
|  | D | 0.09 | 0.08 | -0.07 | 0.24 | 0.27 |
|  | C | 0.06 | 0.08 | -0.09 | 0.21 | 0.41 |
|  | B | 0.11 | 0.08 | -0.14 | 0.16 | 0.89 |
|  | A | Ref | - | - | - | - |
| Current ENDS use | Everyday user | -0.13 | 0.08 | -0.29 | 0.03 | 0.11 |
|  | Someday user | -0.34 | 0.08 | -0.50 | -0.19 | <**0.001** |
|  | Former user | -0.21 | 0.06 | -0.14 | 0.10 | 0.73 |
|  | Never user | Ref | - | - | **-** | - |
| Worry for contracting COVID-19 | More worry | 0.07 | 0.05 | -0.02 | 0.17 | 0.14 |
|  | No or less worry | Ref | - | - | **-** | - |
| Confident of not contracting COVID | Agreeing | 0.07 | 0.05 | -0.03 | 0.16 | 0.17 |
|  | Disagreeing | Ref | - | - | **-** | - |
| Age (yrs.) | 21-30 | Ref | - | - | **-** | - |
|  | 31-40 | -0.07 | 0.05 | -0.17 | 0.03 | 0.16 |
|  | 41-45 | 0.11 | 0.07 | -0.03 | 0.24 | 0.13 |
| Sex | Male | Ref | - | - | **-** | - |
|  | Female | 0.13 | 0.05 | 0.03 | 0.23 | **0.01** |
| Race & ethnicity  (N=604) | Non-Hispanic White individuals | Ref | - | - | **-** | - |
|  | Non-Hispanic Black individuals | 0.01 | 0.07 | -0.13 | 0.14 | 0.92 |
|  | Hispanic individuals | -0.02 | 0.06 | -0.15 | 0.10 | 0.71 |
|  | Others | -0.09 | 0.10 | -0.28 | 0.10 | 0.36 |
| Annual income (US dollars)  (N=604) | <$20,000 | Ref | - | - | **-** | - |
|  | $20,000 to $34,999 | 0.27 | 0.09 | 0.10 | 0.44 | **0.02** |
|  | $35,000 to $49,999 | 0.28 | 0.09 | 0.11 | 0.45 | **0.001** |
|  | $50,000 to $74,999 | 0.30 | 0.09 | 0.13 | 0.46 | **<0.001** |
|  | $75,000 to $99,999 | 0.34 | 0.10 | 0.15 | 0.54 | **<0.001** |
|  | ≥ $100,000 | 0.33 | 0.09 | 0.16 | 0.51 | **<0.001** |
| Education  (N=604) | High school or less | Ref | - | - | **-** | - |
|  | Post-High school | -0.09 | 0.10 | -0.29 | 0.11 | 0.39 |
|  | Some college | -0.15 | 0.07 | -0.29 | -0.01 | **0.04** |
|  | College graduate and post-graduate | -0.15 | 0.07 | -0.29 | -0.01 | **0.04** |

Supplementary table 2: Generalized linear model for intentions to purchase IQOS in next 12 months among current smokers. OR= Odds ratio, CI= Confidence interval.

| Variable name | | B-coefficient | Std. error | 95% CI | | p-value |
| --- | --- | --- | --- | --- | --- | --- |
|  |  |  |  | Lower | Upper |  |
| Product appeal | | 0.09 | 0.01 | 0.07 | 0.11 | **<0.001** |
| Recruitment platform | Prime panel | -0.09 | 0.05 | -0.19 | 0.01 | 0.06 |
|  | Mturk | Ref | - | - | **-** | - |
| Randomized ad condition | F | 0.01 | 0.07 | -0.13 | 0.15 | 0.91 |
|  | E | 0.02 | 0.07 | -0.12 | 0.16 | 0.77 |
|  | D | 0.09 | 0.07 | -0.05 | 0.22 | 0.22 |
|  | C | 0.05 | 0.07 | -0.09 | 0.18 | 0.49 |
|  | B | -0.02 | 0.07 | -0.16 | 0.12 | 0.78 |
|  | A | Ref | - | - | - | - |
| Current ENDS use | Everyday user | -0.12 | 0.08 | -0.27 | 0.03 | 0.11 |
|  | Someday user | 0.07 | 0.07 | -0.08 | 0.22 | 0.36 |
|  | Former user | -0.04 | 0.06 | -0.15 | 0.07 | 0.46 |
|  | Never user | Ref | - | - | **-** | - |
| Worry for contracting COVID-19 | More worry | 0.04 | 0.04 | -0.05 | 0.13 | 0.36 |
|  | No or less worry | Ref | - | - | **-** | - |
| Confident of not contracting COVID | Agreeing | 0.03 | 0.04 | -0.06 | 0.11 | 0.51 |
|  | Disagreeing | Ref | - | - | **-** | - |
| Age (yrs.) | 21-30 | Ref | - | - | **-** | - |
|  | 31-40 | 0.00 | 0.05 | -0.09 | 0.09 | 0.94 |
|  | 41-45 | 0.00 | 0.06 | -0.12 | 0.13 | 0.94 |
| Sex | Male | Ref | - | - | **-** | - |
|  | Female | 0.10 | 0.05 | 0.01 | 0.18 | **0.04** |
| Race & ethnicity  (N=604) | Non-Hispanic White individuals | Ref | - | - | **-** | - |
|  | Non-Hispanic Black individuals | 0.09 | 0.06 | -0.21 | 0.04 | 0.19 |
|  | Hispanic individuals | -0.06 | 0.06 | -0.17 | 0.06 | 0.34 |
|  | Others | -0.04 | 0.09 | -0.21 | 0.13 | 0.64 |
| Annual income (US dollars)  (N=604) | <$20,000 | Ref | - | - | **-** | - |
|  | $20,000 to $34,999 | 0.10 | 0.08 | -0.06 | 0.26 | 0.20 |
|  | $35,000 to $49,999 | 0.16 | 0.08 | 0.01 | 0.31 | **0.05** |
|  | $50,000 to $74,999 | 0.15 | 0.08 | -0.00 | 0.31 | 0.05 |
|  | $75,000 to $99,999 | 0.14 | 0.09 | -0.03 | 0.32 | 0.11 |
|  | ≥ $100,000 | 0.17 | 0.08 | 0.01 | 0.33 | 0.04 |
| Education  (N=604) | High school or less | Ref | - | - | **-** | - |
|  | Post-High school | -0.05 | 0.09 | -0.23 | 0.14 | 0.63 |
|  | Some college | -0.04 | 0.07 | -0.17 | 0.09 | 0.56 |
|  | College graduate and post-graduate | -0.09 | 0.07 | -0.21 | 0.04 | 0.19 |

Supplementary table 3: Generalized linear model for intentions to try IQOS if offered for free among current smokers. OR= Odds ratio, CI= Confidence interval.

| Variable name | | B-coefficient | Std. error | 95% CI | | p-value |
| --- | --- | --- | --- | --- | --- | --- |
|  |  |  |  | Lower | Upper |  |
| Product appeal | | 0.11 | 0.02 | 0.07 | 0.14 | **<0.001** |
| Recruitment platform | Prime panel | 0.19 | 0.12 | -0.04 | 0.43 | 0.11 |
|  | Mturk | Ref | - | - | **-** | - |
| Randomized ad condition | F | -0.33 | 0.17 | -0.68 | 0.01 | 0.06 |
|  | E | -0.02 | 0.16 | -0.33 | 0.28 | 0.88 |
|  | D | -0.27 | 0.13 | -0.52 | -0.01 | 0.04 |
|  | C | 0.03 | 0.13 | -0.22 | 0.27 | 0.84 |
|  | B | -0.06 | 0.15 | -0.36 | 0.24 | 0.71 |
|  | A | Ref | - | - | - | - |
| Current ENDS use | Everyday user | -0.91 | 0.15 | -1.21 | -0.62 | <0.001 |
|  | Someday user | -0.92 | 0.12 | -0.15 | -0.68 | <**0.001** |
|  | Former user | 0.11 | 0.16 | -0.20 | 0.41 | 0.50 |
|  | Never user | Ref | - | - | **-** | - |
| Worry for contracting COVID-19 | More worry | 0.04 | 0.11 | -0.18 | 0.26 | 0.75 |
|  | No or less worry | Ref | - | - | **-** | - |
| Confident of not contracting COVID | Agreeing | 0.27 | 0.13 | 0.02 | 0.52 | **0.03** |
|  | Disagreeing | Ref | - | - | **-** | - |
| Age (yrs.) | 21-30 | Ref | - | - | **-** | - |
|  | 31-40 | -0.04 | 0.11 | -0.24 | 0.17 | 0.73 |
|  | 41-45 | 0.03 | 0.14 | -0.24 | 0.31 | 0.82 |
| Sex | Male | Ref | - | - | **-** | - |
|  | Female | 0.12 | 0.10 | -0.07 | 0.32 | 0.22 |
| Race & ethnicity  (N=604) | Non-Hispanic White individuals | Ref | - | - | **-** | - |
|  | Non-Hispanic Black individuals | -0.03 | 0.19 | -0.40 | 0.35 | 0.89 |
|  | Hispanic individuals | -0.03 | 0.17 | -0.36 | 0.31 | 0.88 |
|  | Others | -0.18 | 0.17 | -0.51 | 0.15 | 0.28 |
| Annual income (US dollars)  (N=604) | <$20,000 | Ref | - | - | **-** | - |
|  | $20,000 to $34,999 | -0.15 | 0.16 | -0.47 | 0.17 | 0.36 |
|  | $35,000 to $49,999 | -0.11 | 0.14 | -0.39 | 0.18 | 0.47 |
|  | $50,000 to $74,999 | 0.07 | 0.13 | -0.18 | 0.32 | 0.57 |
|  | $75,000 to $99,999 | -0.07 | 0.15 | -0.36 | -0.22 | 0.63 |
|  | ≥ $100,000 | 0.32 | 0.16 | 0.01 | 0.63 | **0.04** |
| Education  (N=604) | High school or less | Ref | - | - | **-** | - |
|  | Some college | -0.35 | 0.13 | -0.60 | -0.09 | **0.01** |
|  | College graduate and post-graduate | -0.22 | 0.11 | -0.43 | -0.01 | **0.04** |

Supplementary table 4: Generalized linear model for intentions to purchase IQOS in next 12 months among former smokers. OR= Odds ratio, CI= Confidence interval.

| Variable name | | B-coefficient | Std. error | 95% CI | | p-value |
| --- | --- | --- | --- | --- | --- | --- |
|  |  |  |  | Lower | Upper |  |
| Product appeal | | 0.16 | 0.03 | 0.11 | 0.21 | **<0.001** |
| Recruitment platform | Prime panel | -0.41 | 0.17 | -0.74 | -0.09 | **0.01** |
|  | Mturk | Ref | - | - | **-** | - |
| Randomized ad condition | F | -0.35 | 0.24 | -0.83 | 0.12 | 0.15 |
|  | E | 0.58 | 0.22 | 0.15 | 1.01 | 0.01 |
|  | D | -0.48 | 0.19 | -0.84 | -0.12 | **0.01** |
|  | C | 0.03 | 0.18 | -0.32 | 0.37 | 0.88 |
|  | B | 0.14 | 0.21 | -0.27 | 0.56 | 0.50 |
|  | A | Ref | - | - | - | - |
| Current ENDS use | Everyday user | -0.80 | 0.20 | -1.19 | -0.40 | <0.001 |
|  | Someday user | -0.84 | 0.15 | -1.14 | -0.54 | <0.001 |
|  | Former user | 0.23 | 0.22 | -0.20 | 0.66 | 0.29 |
|  | Never user | Ref | - | - | **-** | - |
| Worry for contracting COVID-19 | More worry | 0.05 | 0.16 | -0.26 | 0.36 | 0.74 |
|  | No or less worry | Ref | - | - | **-** | - |
| Confident of not contracting COVID | Agreeing | 0.60 | 0.18 | 0.24 | 0.95 | **0.001** |
|  | Disagreeing | Ref | - | - | **-** | - |
| Age (yrs.) | 21-30 | Ref | - | - | **-** | - |
|  | 31-40 | -0.25 | 0.15 | -0.54 | 0.05 | 0.10 |
|  | 41-45 | -0.40 | 0.20 | -0.80 | -0.12 | **0.04** |
| Sex | Male | Ref | - | - | **-** | - |
|  | Female | 0.36 | 0.14 | 0.09 | 0.64 | **0.01** |
| Race & ethnicity  (N=604) | Non-Hispanic White individuals | Ref | - | - | **-** | - |
|  | Non-Hispanic Black individuals | -0.38 | 0.28 | -0.91 | 0.16 | 0.17 |
|  | Hispanic individuals | 0.20 | 0.24 | -0.26 | 0.66 | 0.40 |
|  | Others | -0.18 | 0.17 | -0.51 | 0.15 | 0.28 |
| Annual income (US dollars)  (N=604) | <$20,000 | Ref | - | - | **-** | - |
|  | $20,000 to $34,999 | -0.38 | 0.23 | -0.83 | 0.07 | 0.10 |
|  | $35,000 to $49,999 | 0.36 | 0.21 | -0.05 | 0.77 | 0.09 |
|  | $50,000 to $74,999 | -0.08 | 0.18 | -0.43 | 0.27 | 0.65 |
|  | $75,000 to $99,999 | -0.69 | 0.21 | -1.11 | -0.28 | 0.001 |
|  | ≥ $100,000 | 0.04 | 0.22 | -0.39 | 0.48 | 0.85 |
| Education  (N=604) | High school or less | Ref | - | - | **-** | - |
|  | Some college | -0.08 | 0.19 | -0.45 | 0.28 | 0.66 |
|  | College graduate and post-graduate | -0.14 | 0.15 | -0.44 | 0.15 | 0.34 |

Supplementary table 5: Generalized linear model for intentions to try IQOS if offered for free among former smokers. OR= Odds ratio, CI= Confidence interval.

| Variable name | | B-coefficient | Std. error | 95% CI | | p-value |
| --- | --- | --- | --- | --- | --- | --- |
|  |  |  |  | Lower | Upper |  |
| Product appeal | | 0.16 | 0.02 | 0.13 | 0.19 | **<0.001** |
| Recruitment platform | Prime panel | 0.29 | 0.12 | 0.06 | 0.52 | **0.02** |
|  | Mturk | Ref | - | - | **-** | - |
| Randomized ad condition | F | -0.11 | 0.16 | -0.43 | 0.21 | 0.49 |
|  | E | -0.08 | 0.15 | -0.39 | 0.22 | 0.58 |
|  | D | 0.07 | 0.17 | -0.27 | -0.41 | 0.69 |
|  | C | 0.01 | 0.16 | -0.29 | 0.32 | 0.94 |
|  | B | -0.15 | 0.15 | -0.44 | 0.14 | 0.31 |
|  | A | Ref | - | - | - | - |
| Current ENDS use | Everyday user | 0.93 | 0.33 | 0.28 | 1.58 | 0.01 |
|  | Someday user | 0.58 | 0.16 | 0.27 | 0.89 | <**0.001** |
|  | Former user | 0.05 | 0.13 | -0.20 | 0.30 | 0.67 |
|  | Never user | Ref | - | - | **-** | - |
| Worry for contracting COVID-19 | More worry | 0.08 | 0.11 | -0.12 | 0.29 | 0.42 |
|  | No or less worry | Ref | - | - | **-** | - |
| Confident of not contracting COVID | Agreeing | 0.22 | 0.11 | 0.01 | 0.43 | **0.04** |
|  | Disagreeing | Ref | - | - | **-** | - |
| Age (yrs.) | 18-20 | Ref | - | - | **-** | - |
|  | 21-30 | 0.08 | 0.23 | -0.38 | 0.53 | 0.74 |
|  | 31-40 | 0.06 | 0.24 | -0.40 | 0.53 | 0.90 |
|  | 41-45 | 0.03 | 0.26 | -0.48 | 0.55 | 0.90 |
| Sex | Male | Ref | - | - | **-** | - |
|  | Female | -0.13 | 0.10 | -0.33 | 0.08 | 0.23 |
| Race & ethnicity  (N=604) | Non-Hispanic White individuals | Ref | - | - | **-** | - |
|  | Non-Hispanic Black individuals | -0.23 | 0.13 | -0.49 | 0.03 | 0.08 |
|  | Hispanic individuals | -0.06 | 0.15 | -0.35 | 0.24 | 0.71 |
|  | Others | -0.11 | 0.13 | -0.37 | 0.15 | 0.40 |
| Annual income (US dollars)  (N=604) | <$20,000 | Ref | - | - | **-** | - |
|  | $20,000 to $34,999 | -0.03 | 0.17 | -0.31 | 0.36 | 0.88 |
|  | $35,000 to $49,999 | -0.07 | 0.16 | -0.37 | 0.23 | 0.65 |
|  | $50,000 to $74,999 | 0.23 | 0.14 | -0.05 | 0.50 | 0.11 |
|  | $75,000 to $99,999 | 0.25 | 0.16 | -0.05 | 0.56 | 0.11 |
|  | ≥ $100,000 | -0.11 | 0.15 | -0.39 | 0.18 | 0.48 |
| Education  (N=604) | High school or less | Ref | - | - | **-** | - |
|  | Some college | 0.13 | 0.30 | -0.45 | 0.71 | 0.67 |
|  | College graduate and post-graduate | -0.20 | 0.15 | -0.49 | 0.08 | 0.16 |

Supplementary table 6: Generalized linear model for intentions to purchase IQOS in next 12 months among never smokers. OR= Odds ratio, CI= Confidence interval.

| Variable name | | B-coefficient | Std. error | 95% CI | | p-value |
| --- | --- | --- | --- | --- | --- | --- |
|  |  |  |  | Lower | Upper |  |
| Product appeal | | 0.18 | 0.01 | 0.15 | 0.21 | **<0.001** |
| Recruitment platform | Prime panel | 0.29 | 0.11 | 0.07 | 0.50 | 0.01 |
|  | Mturk | Ref | - | - | **-** | - |
| Randomized ad condition | F | -0.12 | 0.15 | -0.42 | 0.17 | 0.41 |
|  | E | -0.15 | 0.14 | -0.43 | 0.14 | 0.31 |
|  | D | 0.00 | 0.16 | -0.31 | -0.31 | 1.00 |
|  | C | 0.09 | 0.14 | -0.19 | 0.37 | 0.52 |
|  | B | -0.22 | 0.14 | -0.49 | 0.05 | 0.11 |
|  | A | Ref | - | - | - | - |
| Current ENDS use | Everyday user | 0.63 | 0.32 | 0.01 | 1.25 | 0.05 |
|  | Someday user | 0.68 | 0.15 | 0.39 | 0.98 | <**0.001** |
|  | Former user | 0.38 | 0.12 | 0.15 | 0.61 | **0.001** |
|  | Never user | Ref | - | - | **-** | - |
| Worry for contracting COVID-19 | More worry | -0.00 | 0.10 | -0.20 | 0.19 | 1.00 |
|  | No or less worry | Ref | - | - | **-** | - |
| Confident of not contracting COVID | Agreeing | 0.07 | 0.10 | -0.12 | 0.26 | 0.49 |
|  | Disagreeing | Ref | - | - | **-** | - |
| Age (yrs.) | 18-20 | Ref | - | - | **-** | - |
|  | 21-30 | 0.11 | 0.22 | -0.33 | 0.55 | 0.63 |
|  | 31-40 | 0.22 | 0.23 | -0.22 | 0.66 | 0.33 |
|  | 41-45 | 0.18 | 0.25 | -0.30 | 0.66 | 0.47 |
| Sex | Male | Ref | - | - | **-** | - |
|  | Female | -0.15 | 0.10 | -0.34 | 0.04 | 0.12 |
| Race & ethnicity  (N=604) | Non-Hispanic White individuals | Ref | - | - | **-** | - |
|  | Non-Hispanic Black individuals | -0.19 | 0.12 | -0.43 | 0.05 | 0.13 |
|  | Hispanic individuals | -0.00 | 0.14 | -0.28 | 0.27 | 0.99 |
|  | Others | -0.23 | 0.12 | -0.48 | 0.01 | 0.06 |
| Annual income (US dollars)  (N=604) | <$20,000 | Ref | - | - | **-** | - |
|  | $20,000 to $34,999 | 0.06 | 0.16 | -0.25 | 0.38 | 0.69 |
|  | $35,000 to $49,999 | -0.25 | 0.15 | -0.54 | 0.04 | 0.09 |
|  | $50,000 to $74,999 | 0.07 | 0.13 | -0.19 | 0.33 | 0.62 |
|  | $75,000 to $99,999 | 0.03 | 0.15 | -0.26 | 0.32 | 0.84 |
|  | ≥ $100,000 | -0.30 | 0.14 | -0.57 | 0.02 | 0.04 |
| Education  (N=604) | High school or less | Ref | - | - | **-** | - |
|  | Some college | 0.31 | 0.28 | -0.24 | 0.85 | 0.27 |
|  | College graduate and post-graduate | 0.05 | 0.14 | -0.22 | 0.32 | 0.71 |

Supplementary table 7: Generalized linear model for intentions to try IQOS if offered for free among never smokers. OR= Odds ratio, CI= Confidence interval.
